# Efficacy of lotilaner against myiasis caused by *Cochliomyia hominivorax* (Diptera: Calliphoridae) in naturally infested dogs

**DOI:** 10.1186/s13071-023-05661-z

**Published:** 2023-03-06

**Authors:** Tássia Lopes do Vale, Alcyjara Rego Costa, Leandro Macedo Miranda, Geovane Ferreira Silva, Naylene Carvalho Sales Silva, Tiago Barbalho Lima, Daniel Praseres Chaves, Heinz Sager, Pedro Veloso Facury Lasmar, Livio Martins Costa–Junior

**Affiliations:** 1 Postgraduate Program in Health Sciences, Federal University of Maranhão (UFMA), São Luís, MA Brazil; 2 Department of Pathology, State University of Maranhão (UEMA), São Luís, MA Brazil; 3 Department of Pathology, Federal University of Maranhão (UFMA), São Luís, MA Brazil; 4 Elanco Animal Health Inc, Basel, Switzerland; 5 Elanco Animal Health Inc, São Paulo, Brazil

**Keywords:** New World screwworm fly, Ectoparasites, Dogs, Myiasis, Control strategies

## Abstract

**Background:**

The New World screwworm fly, *Cochliomyia hominivorax*, is widely distributed across South America. This parasitic insect is a significant cause of primary myiasis in animals, including dogs. There is an urgent need for a rapid and efficient treatment to improve the recovery of affected animals. In the present study we evaluated the potential of lotilaner for the treatment of myiasis caused by *C. hominivorax* larvae in naturally infested dogs. Lotilaner belongs to the isoxazoline class of chemical compounds and is marketed as Credelio™ for use against ticks and fleas in dogs and cats.

**Methods:**

Eleven dogs with naturally acquired myiasis were enrolled in this study based on the severity of lesions and the number of identified larvae. All animals received a single oral administration of lotilaner at a minimum dose of 20.5 mg/kg body weight. After treatment, the number of expelled larvae, live or dead, was determined at 2, 6 and 24 h, and the larval expulsion rate, larvicidal effect and overall efficacy were calculated. After 24 h, the remaining larvae were removed, counted and identified. The lesions were cleaned, and palliative treatment was administered when necessary, according to the animal's health status.

**Results:**

All larvae were identified as *C. hominivorax*. The larval expulsion rate was 80.5% and 93.0% at 2 and 6 h post-treatment, respectively. Lotilaner showed an overall efficacy of 100% at 24 h post-treatment.

**Conclusions:**

Lotilaner demonstrated a rapid onset of action and a high efficacy against *C. hominivorax*. We therefore recommend lotilaner for the effective treatment of myiasis in dogs.

**Graphical Abstract:**

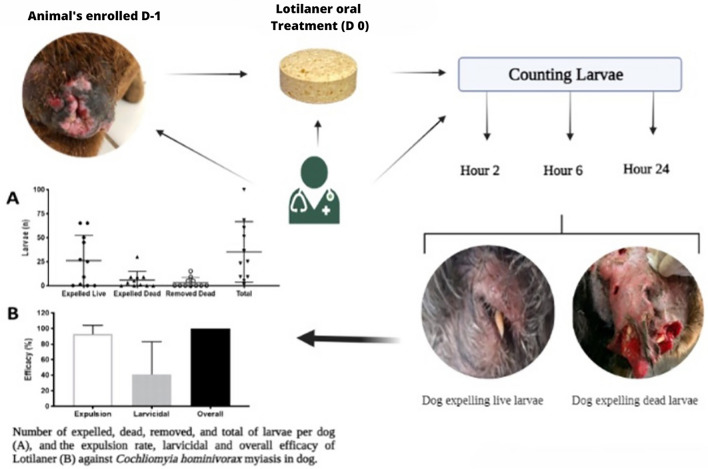

## Background

Myiasis is a disease caused by the larval stages of several species of flies that feed on tissue from the hosts. However, in South America, including Brazil, the New World screwworm (NWS), *Cochliomyia hominivorax* (Diptera: Calliphoridae), is the main species causing primary myiasis in humans and warm-blooded animals [1, 2]. Historically, the geographic distribution of NWS extended from the southern USA to the central region of Argentina [3]. In the late 1960s, the USA successfully launched a program aimed at eradication of this species by releasing sterile insects within its national borders, later expanding the program to Mexico and finally to continental Central America [4]. Panama currently represents the biological barrier, achieved by releasing sterile insects within its territory along the border with Colombia [5]. As a result of these eradication programs, the current distribution of this parasitic fly comprises the Caribbean and South American countries, with the exception of Chile. There is a proposal for an eradication program in Uruguay which, if implemented, will require constant epidemiological surveillance on the border with Brazil and Argentina [6].

To eradicate *C. hominivorax* and eliminate the need for epidemiological surveillance, it is necessary, in addition to the massive release of sterile insects, to diagnose and treat all cases of myiasis in a given area [4, 7]. In companion animals, NWS myiasis is a debilitating disease and can be fatal depending on the time to diagnosis and treatment, level of infestation and the site of infestation [8]. The threat of reinvasion of the NWS in areas where it has been eradicated increases proportionally with increasing international animal trade and the travel of pets and humans. Dogs are important hosts for NWS, and the diagnosis and treatment of myiasis are essential for effective control of this disease, as described for the outbreaks in Florida (USA) in 2016 [9]. Control measures are even more important in the context of the One Health perspective and the fact that NWS is not limited to domestic animals [10].

In this context, a rapid and efficient treatment is necessary to improve the recovery of affected animals and reduce the possibility of reinvasion in the areas where this parasitic fly has been eradicated. Various topical and systemic drugs are used to treat myiasis in dogs (e.g. macrocyclic lactones, nitenpyram and spinosad) [8, 11–13]. More recently, other parasiticides, such as isoxazolines, have been described for the effective treatment of cutaneous myiasis [11, 14, 15].

The isoxazoline parasiticide lotilaner acts as a potent non-competitive antagonist of GABA-activated chloride channels from arthropods [16]. Lotilaner has been registered for use in dogs and cats in a chewable tablet-formulation as Credelio™ (Elanco Animal Health, Indianapolis, IN, USA) and demonstrated efficacy against ticks [17–19], mange mite [20] and fleas [21, 22]. The efficacy of lotilaner against myiasis by the Old-World screwworm (*Chrysomya bezziana*) was recently shown in two cats from Malaysia [23]. Although the action of isoxazolines on *C. hominivorax* has been documented, the efficacy of lotilaner on myiasis caused by this parasite has not yet been shown. The objective of the present work was to verify the potential of lotilaner for the treatment of naturally acquired screwworm myiasis caused by *C. hominivorax* in dogs.

## Methods

The experimental procedures were approved by the animal research ethics committee of the Federal University of Maranhão—UFMA (CIAEP: 02.0341.2019), Brazil, under protocol number 23115.005441/2017-62.

### Experimental design

Eleven client-owned dogs (5 males, 6 females) that were naturally infested with *C. hominivorax* and with active myiasis were enrolled in the study. All larvae collected during the experiment were maintained in 70% ethanol and identified as *C. hominivorax,* according to Stojanovich et al. [24]. The age of the enrolled dogs ranged from 1.5 to 10.0 years and with body weight ranged between 3.3 and 25.0 kg. The dogs had not received any ectoparasiticide treatment in the 120 days immediately preceding the experiment. The myiasis lesions were distributed in different body areas: mammary (3 dogs), eye (3 dogs), scrotum (2 dogs), neck and thoracic and pelvic limbs (1 dog each).

After myiasis had been diagnosed, based on the observation of larvae in the wound, the dogs received lotilaner (Credelio™) in a single dose orally, following the manufacturer's recommended dose for control of fleas and ticks in dogs. The doses ranged from 23.9 to 40.9 mg/kg body weight. After treatment, the dogs were kept in individual kennels with a removable tray (surface area of 0.3–0.8 m^2^ according to body weight). The dogs were observed at 2 and 6 h post-treatment at which times expelled larvae were collected and quantified. At 24 h post-treatment, the remaining larvae were mechanically removed from the wound. Larvae without movement were considered to be dead. After the removal of the remaining larvae, the wounds were cleaned and an anti-inflammatory (0.2 mg/kg of meloxicam, applied subcutaneously [SC]) and antibiotic (15.0 mg/kg of amoxicillin trihydrate, SC) treatment was administered before the dogs were sent home. The dogs were evaluated at home daily for general health conditions, and the wounds were cleaned by the owners until complete healing had been achieved.

### Data analysis

The evaluation of the efficacy of lotilaner against *C. hominivorax* was calculated based on the formulae described by Oliveira et al. [13]. The overall efficacy was calculated as: [(number of dead larvae expelled + number of live larvae expelled + number of dead larvae removed)/total number of larvae] × 100. The larval expulsion efficacy was calculated at 2, 6 and 24 h post-treatment using the formula: [(number of dead larvae expelled + number of live larvae expelled)/total number of larvae] × 100. The larvicidal efficacy was calculated using the formula: [(number of dead larvae expelled + number of dead larvae removed)/total number of larvae] × 100.

## Results

**Fig. 1:**
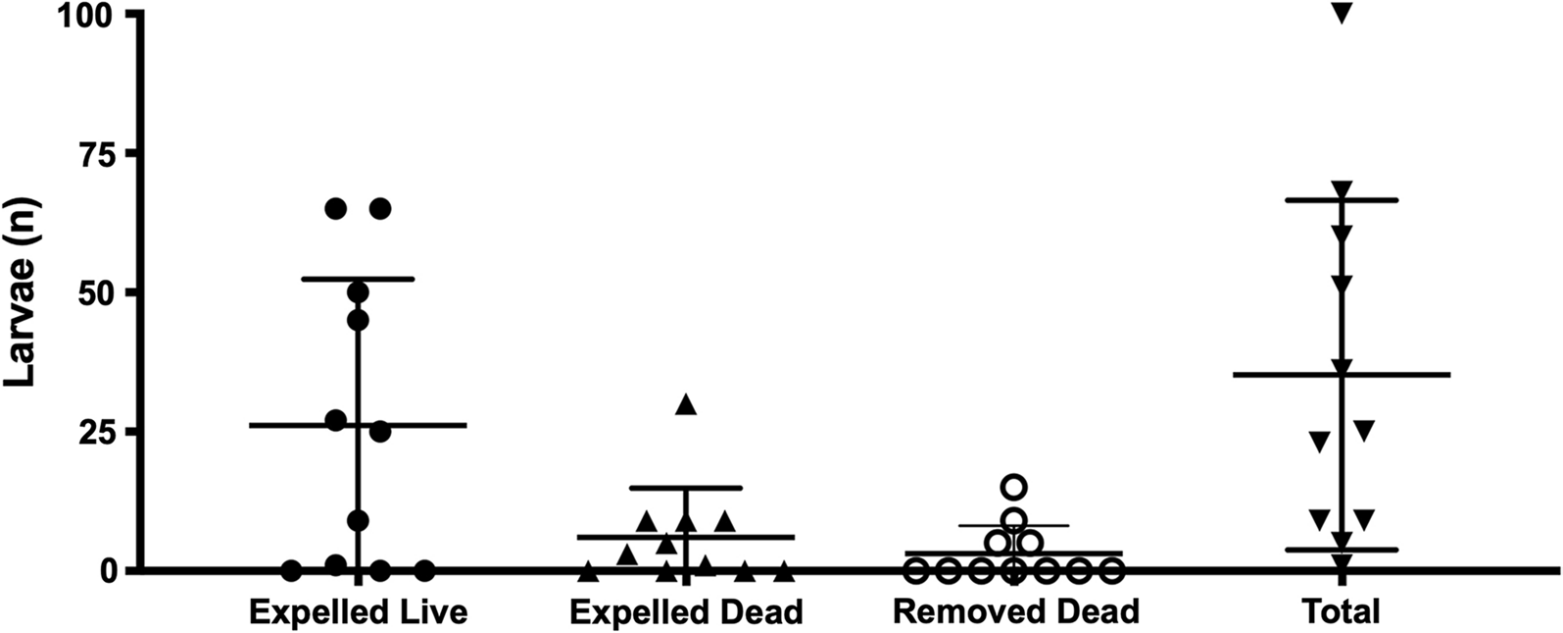
Mean and standard deviation of the number of expelled, dead, removed and total number of larvae of *Cochliomyia hominivorax* per dog treated with a single oral dose of Credelio™ (lotilaner)

All larvae collected during the study were confirmed as *C. hominivorax*. No adverse effects related to lotilaner or any other treatment were observed throughout the study. Each dog had only a single wound with active myiasis and an average of 35 larvae (range: 1–100) (Fig. 1).

**Fig. 2:**
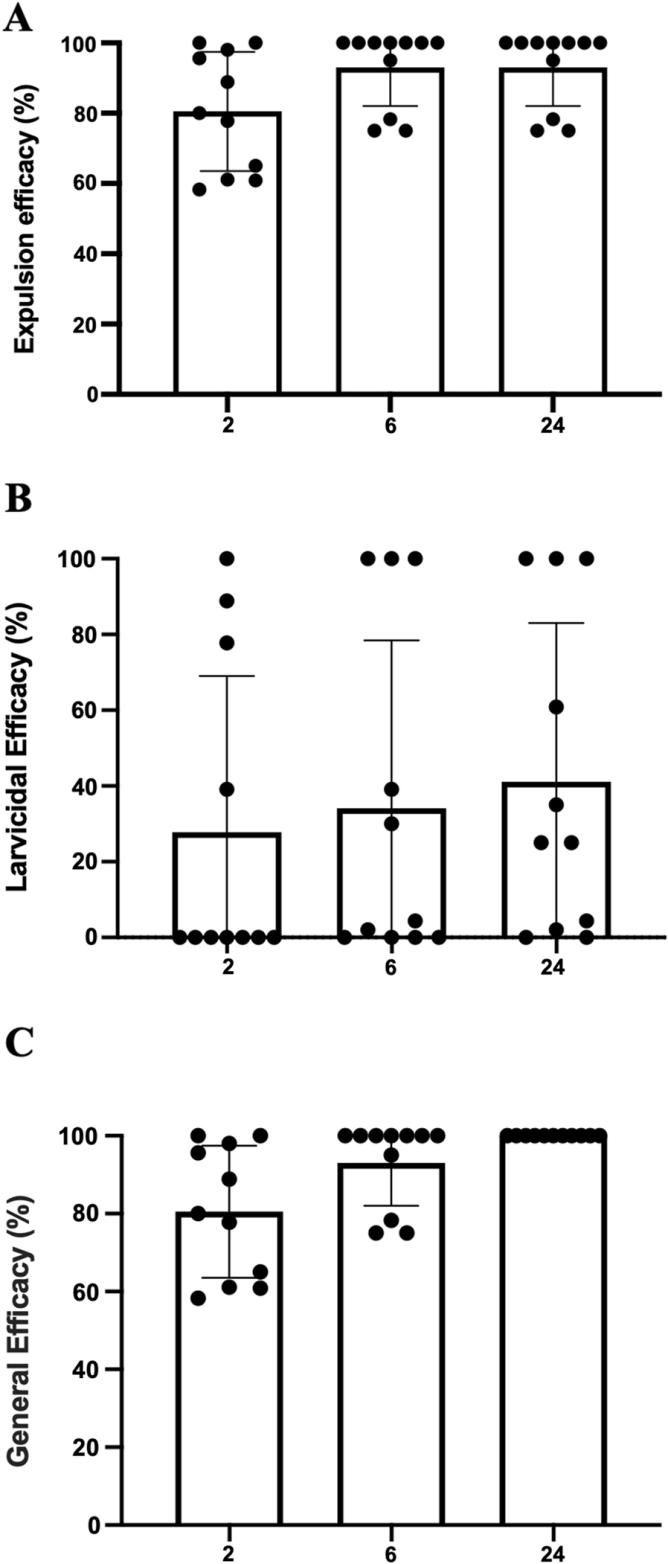
Expulsion (**a**), larvicidal efficacy (**b**) and general efficacy (**c**) of a single dose of Credelio™ (lotilaner) in dogs at 2, 6 and 24 h post-treatment on myiasis by *Cochliomyia hominivorax*. Dots show the individual values for each dog, the bar shows the average value and the horizontal lines show the standard deviation

A rapid onset of activity was observed, with highest mean expulsion of larvae at 2 h (80.5%), which increased up to 93.0% at 24 h post-treatment (Fig. 2a). Expulsed larvae comprised both live and dead larvae (Fig. 1). Larvae that were removed from the wounds 24 h post-treatment were all dead, and the overall efficacy was 100% at that time point (Fig. 2c).

The mean larvicidal efficacy of lotilaner was rather low at 41.1% at 24 h post-treatment (Fig. 2b), which suggests that the efficacy of the treatment was mostly driven by larval expulsion. The combination of larva expulsion and the larvicidal effect results in a high cumulative efficacy of 80.5%, 93.0% and 100.0% at 2, 6, and 24 h post-treatment, respectively (Fig. 2c).

## Discussion

Myiasis is an infestation of live tissue by the larvae of several species of flies [25]. Larvae of *C. hominivorax* are the main cause of primary myiasis, which represents one of the major skin diseases in dogs in areas where this fly species is present [1, 26]. The number of wounds and larvae in each dog enrolled in the present study was similar to that reported in earlier studies in other regions of Brazil [27]. The highest prevalence of myiasis in Brazil has been reported to be in adult dogs, with no gender predilection [27, 28]. These earlier reports corroborate the observations made on the dogs enrolled in the present study.

Topical insecticides in ointment or spray formulations were the main treatments for myiasis before effective systemic compounds became commercially available. Lotilaner belongs to the isoxazoline chemical class, and chemical compounds in this class are the most recommended for use in Brazil for the control of ectoparasites [29]. The oral administration of a drug has the advantage of reduced exposure risks for pet owners and the environment, compared to topical administration. At the same time, it is expected that used compounds used lead to fast elimination of the larvae. The rapid activity of lotilaner and high larval expulsion at 2 h post-treatment, as shown in the present study, can be explained by the early peaking of lotilaner concentration in the blood within 2 h after treatment [30]. This result is in agreement with those from earlier efficacy studies against fleas, where 64.0% of adult *Ctenocephalides felis* were eliminated 2 h after treatment [21]. Insecticidal compounds that have a rapid onset of efficacy are preferred options for treating primary myiasis in dogs as they allow quick restoration of quality of life [15]. Therefore, a high and rapid expulsion of larvae is important, as it can promote faster healing of lesions and prevent animals from having to undergo procedures such as sedation and prolonged antibiotic therapy. Furthermore, the rapid expulsion of larvae from the host tissue can reduce the risk of foreign body infection and the need to debride the lesion to remove the larvae [23]. Given the lack of guidance for treating myiasis in dogs, the use of systemic molecules should be recommended due to the above-mentioned advantages [14, 15].

Sarolaner, also an isoxazoline, was reported to achieve a similar high cumulative larva expulsion of *C. hominivorax* [15], showing that the efficacy against this parasite could be inherent to all registered compounds of this chemical class. Nitenpyram, a neonicotinoid, also shows a high larval expulsion of *C. hominivorax* after two treatments within 24 h [8], while a single administration of spinosad promoted lower larval expulsion, similar to a single treatment of nitenpyram [13].

Residual protection for at least 30 days against subsequent infestation by *C. hominivorax* should be the focus of further studies. Lotilaner has a terminal half-life of 30.7 days [30] and high insecticide and acaricide efficacy at 30 days of the treatment [18, 22, 31], suggesting that the residual protection against NWS can be expected for the same period of time. Drugs with these characteristics that promote the prevention and control of NWS must be a priority of One Health. Therefore, it is important to encourage the registration of new drugs against *C. hominivorax* and to urge the standardization of protocols for treatment and prevention of myiasis in dogs.

## Conclusion

The results of this study show a rapid onset of action and high efficacy against *C. hominivorax*. In conclusion, lotilaner can be recommended for the effective treatment of myiasis in dogs.

## Data Availability

Data supporting the conclusions of this article are included within the article.
